# Two Symmetrical Maxillary First Molars with Two Disto-Buccal Root Canals

**Published:** 2006-07-01

**Authors:** Jamileh Ghoddusi, Maryam Javidi, Mahdi Vatanpour

**Affiliations:** 1 *Department of Endodontics, Dental Research Center, Faculty of Dentistry, Mashad University of Medical Sciences, Mashad, Iran*; 2 *Department of Endodontics, Faculty of Dentistry, Mashad University of Medical Sciences, Mashad, Iran*

**Keywords:** First Molar, Five Root Canals, Maxillary, Two Disto-Buccal

## Abstract

**INTRODUCTION:** This article describes the diagnosis and treatment of two symmetrical maxillary first molars with two canals in their distobuccal roots. A 32-year-old female had an emergency treatment because of the pain in tooth #16. On the second visit after accessing the pulp chamber and detecting three major canals (MB, DB, and P) and exploring the 4th canal (MB_2_), we noted that there was a 5^th^ canal in the distobuccal root. Also, necrosis was found in the same patient after examining the tooth #26 which was severely decayed. Then after preparing the access cavity, the same order of orifices was found in tooth #16. An electronic apex locator was utilized to ensure that a perforation of the pulpal floor had not occurred. Working films confirmed the presence of an additional canal in distobuccal root.

## INTRODUCTION

The goal of root canal treatment is to clean the root canal system as thoroughly as possible and to fill it in all dimensions (1).

Perhaps, the first maxillary molar and its mesiobuccal anatomy have been studied more than the other teeth. Weine et al. reported that the mesiobuccal root of the maxillary first molar looks very slender in mesiodistal view. However, it appears very broad in a faciolingual direction of a radiographic image (2). Cohen and Bums called this tooth the most treated but least understood posterior tooth with the highest endodontic failure rate (3).

Weine believed many treatment failures in the maxillary permanent first molar were related to not locating and cleaning the mesiolingual (MB_2_) canal. Despite the joining of these two canals in 86% of the cases in 1 to 4 mm short of the apex, in 4% of cases they have two separate apical foramens (2).

The incidence of a mesiolingual canal has been reported as 18.6% by Hartwell and Bellizi (4) and as 96.1% by Kulid and Peters (5). Also Wiene et al. (2), Seidberg et al. (6) and Vertucci (7) in different papers reported this range between 33% and 62%.David et al. have reported a case of a maxillary first molar with three canals in the mesiobuccal root (8). Maggiore presented a case with six canals (P1, P2, P3, MB1, MB2, DB) (9). Other investigators also have presented some cases with different configurations in root canal system of the maxillary first molar (10-18)(Table 1).

A variety of methods have been suggested in locating excessive canals, Neaverth et al. advocated using a heart-shaped access and countersinking the floor of the pulp chamber with a round bur (19). Hartwell and Weller confirmed the need for improved access (20). Pomeranz and Fishelberg discussed the importance of improved access and thoroughly probing the fissure or groove between the major canals (21). The introduction and wide­ spread use of the operating microscope has certainly aided in locating the orifices of the canals. This article describes a patient with two five-canal first maxillary molars that have received successful endodontic treatment.

**Table 1:** Reported canal configuration

**Year**	**Author**	**Canal con ** **figuration**		
		**P**	**MB**	**DB**
1979	Slowey	2P SJ ^a^	1MB	1DB
1979	Thews	2P	1	1
1982	Cecic	2	2	1
1983	Martinez	1	3	2SJ
1984	Beatty	1	3	1
1988	Bond	2J	2S	2J
1991	Wong	3	1	1
1994	Jacobsen	2	1	1
1997	Hulsmann	1	1	2S
2002	Maggior	3S	2S	1
2005	Ferguson	2P	3	l

*J* ^a^ *: Joined canals in apical portion*

*S* ^a^ *: Separated canals*

## CASE REPORT

**Figure 1:**
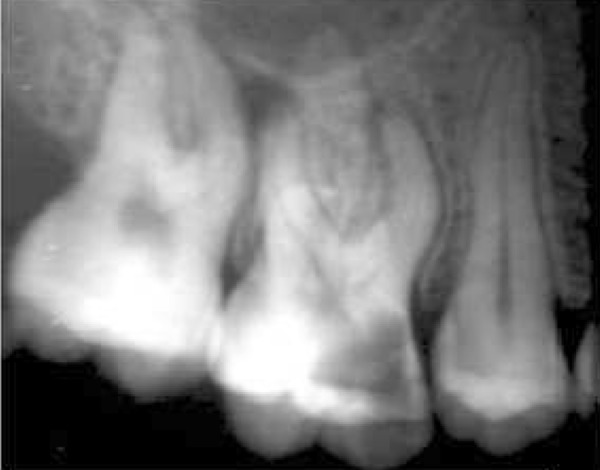
The initial radiograph of tooth #16

A 32-year-old female was referred to the dental clinic of Mashad University of Medical Science, with the chief complaint of a toothache in her right maxilla. Due to deep decay in tooth #16, a diagnosis of symptomatic irreversible pulpitis with a normal periapex was made after vitality testing (Figure 1). Her medical history was unremarkable. Also the involved tooth had class1 Mobility and the probing depths were less than 3 mm.

**Figure 2:**
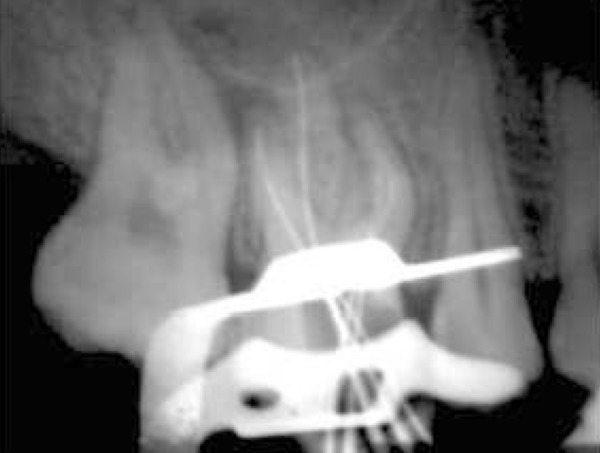
The working length radiograph of tooth #16

**Figure 3:**
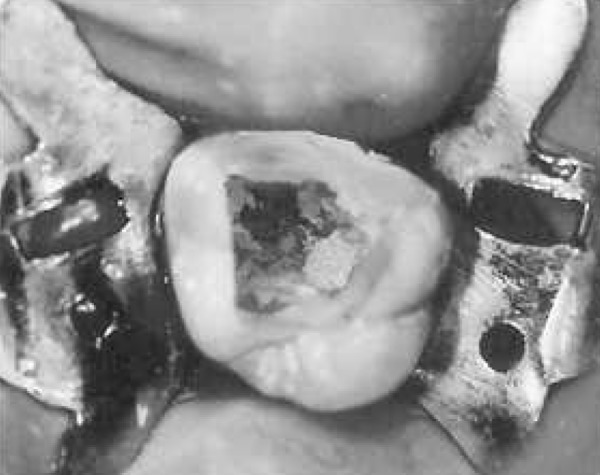
The access cavity following obturation the root canals of tooth #16

**Figure 4:**
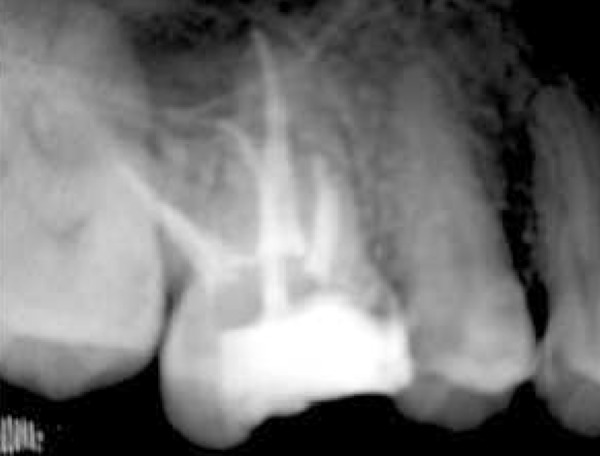
The final radiograph of tooth #16

**Figure 5:**
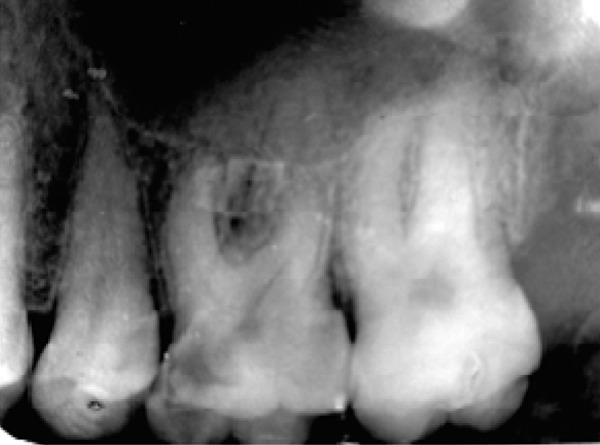
The initial radiograph of tooth #26

After anesthesia (lidocaine plus epinephrine 1:100000) a rubber dam was placed and access cavity was prepared. Following negotiation of the major orifices of canals (P, MB1, DB1), a thorough probing of the fissure between these orifices was performed and MB2 orifice was found in its routine location (Figure 2). Then the initial files #10, 15 (mailefer) were placed in these canals. A working film was exposed and instrumentation was done. Files tended to penetrate another soft area as instrumentation was continued. With a complete exploration of this point and by placing a file # 8, a second working film was exposed and the existence of a canal from a probable perforation was differentiated. It appeared that this canal was in distobuccal root (DB2). Cleaning and shaping was carried out with Race rotary system and then obturation was done with gutta-percha and AH26 (detroy, Densply, USA) as sealer using lateral condensation technique (Figure 3). Crown restoration with amalgam was done. Post operative radiographs revealed a separate canal very near to the distobuccal major canal (Figure 4). Also after examining and testing tooth #26 that had severe decay, it was revealed that it was necrotic and needed root canal treatment (Figure 5).

**Figure 6:**
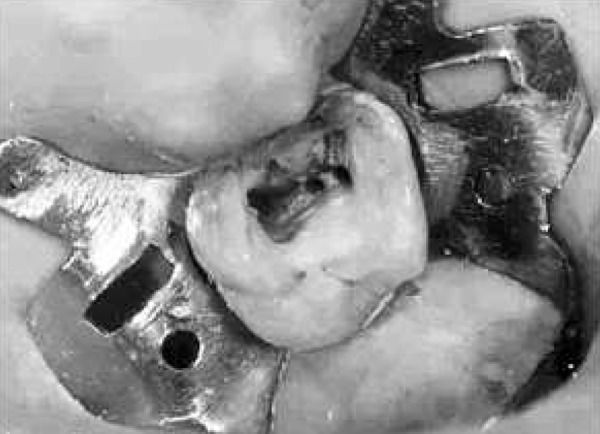
The access cavity of tooth #26

**Figure 7:**
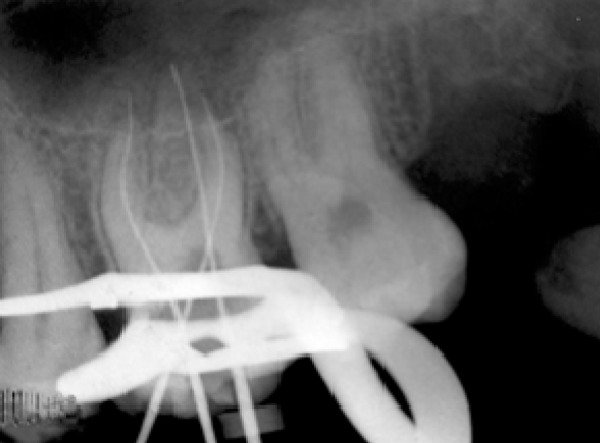
The WL radiograph of tooth #26

On another visit after anesthesia and placing a rubber dam, all caries was removed and the access cavity was prepared (Figure 6). The major canals (P, MB, DBl) was found immediately (Figure 7). Because of the probability and expectation of the existence of other canals, an extended access was prepared and a thorough probing of the fissure between 3 major canals was carried out. Initially, we could locate an orifice between DB, and a palatal canal that was very near to distobuccal canal. We then found a soft area with a different shade. After probing the area with a #2 muller bur and slow speed hand piece, a file could be placed into the area with additional files also placed in the other canals.

**Figure 8:**
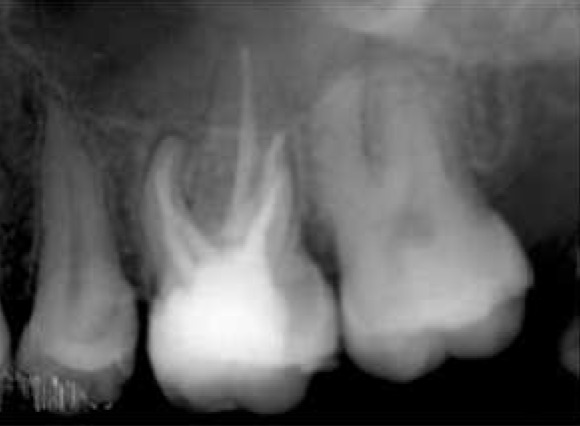
The final radiograph of tooth #26

A working film was exposed and the existences of 5 separate canals were confirmed. Instrumentation was continued. Cleaning and shaping process was completed with NiTi Rotary Race system. Then obturation with gutta-percha and AH26 (Detray, Densply, USA) as a sealer using lateral condensation technique was carried out (Figure 8).

Final working radiographs showed five­ separate canals. Clinically their order appeared similar to an inverted bowling pin. The patient will be monitored clinically and radiographically to ensure a successful treatment.

## DISCUSSION

This article reported an extra-canal in symmetrical maxillary first molars of a patient that was located only by using an extended access and by probing the fissure between the major orifices without the aid of the surgical operating microscope as reported by Fergusen (8).

Therefore it was concluded that an exact radiographic examination and thorough probing of the fissure between the orifices of canals can help us to find most canals and to achieve a more accessible and successful treatment. This was also confirmed by Hartwell and Waller (20).

The young age of the patient involved in this case could have played an important role in the relatively easy identification of the fifth canal without the use of a surgical microscope. It seemed clear that the use of magnification was helpful toward successful treatment, especially in elderly.
